# Platelet Function, Platelet Size and Content of Reticulated Platelets: Interactions in Patients Receiving Dual Antiplatelet Therapy

**DOI:** 10.3390/cells13201712

**Published:** 2024-10-16

**Authors:** Valeria V. Bodrova, Olga N. Shustova, Nina V. Golubeva, Amina K. Alieva, Vladislav V. Vlodzyanovsky, Dmitry V. Pevzner, Alexey V. Mazurov

**Affiliations:** Chazov National Medical Research Center of Cardiology, Russian Ministry of Health, 15a, Academician Chazov Str., Moscow 121552, Russia; malysheva-valeri@mail.ru (V.V.B.); vesta21@yandex.ru (O.N.S.); ngolubeva0@gmail.com (N.V.G.); amina_alieva_1998@mail.ru (A.K.A.); vlodzyanovsky@rambler.ru (V.V.V.); pevsner@mail.ru (D.V.P.)

**Keywords:** platelet function, glycoprotein IIb-IIIa, P-selectin, platelet size, reticulated platelets, antiplatelet drugs, acetylsalicylic acid, clopidogrel, ticagrelor, interleukin 6

## Abstract

Increased platelet activity is a risk factor of thrombotic events in cardiovascular patients. We studied the relationship between platelet function, platelet size, and the content of reticulated platelets (RP) in patients with coronary heart disease (CHD, n = 55) and acute coronary syndrome (ACS, n = 95) receiving acetylsalicylic acid + clopidogrel or ticagrelor, respectively. The control group consisted of patients with risk factors for CHD, but with no CHD/ACS and free of antiplatelet drugs (n = 66). Platelet function was evaluated by the exposure of activated glycoprotein (GP) IIb-IIIa and P-selectin. In the control group, platelets were activated by TRAP (Thrombin Receptor Activating Peptide) 10 µM, and ADP 20, 5, 2.5 µM, and in the CHD/ACS groups, by TRAP 10 µM, and ADP 20 5 µM (±epinephrine 20 µM). Platelet size was assessed by the mean volume, % large forms, and forward scattering. RP were stained by thiazole orange. In the control group, activated GP IIb-IIIa and P-selectin correlated with platelet size and RP content after platelet activation by all agonists. Despite the decrease in platelet activity by antiplatelet drugs, most correlations (primarily for activated GP IIb-IIIa) were preserved in the CHD/ACS patients. In conclusion, increased platelet size and RP content are associated with increased platelet activity and the reduced efficacy of antiplatelet therapy.

## 1. Introduction

Platelet functional activity varies in healthy subjects and patients with cardiovascular diseases receiving antiplatelet therapy [1,2,3,4,5,6,7]. Increased platelet activity is a risk factor of thrombotic events, including myocardial infarction and unstable angina (acute coronary syndrome, ACS), being associated with low sensitivity to antiplatelet drugs [4,5,6,7]. Platelet activity can be affected by platelet phenotypic features such as their size [8,9,10] and the content of “young” reticulated forms [8,10,11].

Early experiments on platelet fractionation and flow cytometry have shown that large platelets have a higher functional activity: they aggregate better, express more adhesive molecules (glycoproteins (GP) IIb-IIIa and Ib), contain more intracellular granules, and produce greater amounts of thromboxane A2 [9,10,12,13,14,15,16]. In both experimental and clinical studies, platelet size is usually assessed using indexes such as mean platelet volume (MPV), platelet large cell ratio (PLC-R), which is measured in impedance-based hematological analyzers, and platelet forward scattering (FSC), which is measured in flow cytometers [9,10,17].

Reticulated platelets (RP) (another term for immature platelets) are “young” platelet forms, recently released into the bloodstream from the bone marrow. They contain residual RNA derived from megakaryocytes, which is destroyed in circulation (platelets do not have nuclei and are unable to synthesize RNA de novo). RP are identified in flow cytometry using RNA-specific fluorescent dyes, such as thiazole orange (TO) and some others. RP represent a minor platelet subpopulation, on average, from 3–5% to about 10% in healthy donors (depending on the detection methods) [10,11]. Microscopic and flow cytometry studies have shown that RP have a larger size [10,11,14,15,18,19,20] and higher functional activity than non-reticulated platelets [10,11,14,18,19,20,21,22,23]. RP have an increased ability for aggregation [18,22,23], contain a higher number of granules [20], and expose more activation markers (activated GP IIb-IIIa and P-selectin) after stimulation by different agonists [14,19,20,21]. RP content correlates with the platelet size of the whole platelet population in both healthy subjects [18,19] and cardiovascular patients treated with antiplatelet drugs [21,24].

In our previous study, we have shown that in healthy subjects increased platelet size and RP content are associated with high platelet activity measured by the activation-dependent exposure of activated GP IIb-IIIa (fibrinogen receptor) and P-selectin (a marker of alpha-granule membranes) using flow cytometry [19]. The binding of activated GP IIb-IIIa with fibrinogen mediates platelet aggregation and P-selectin is involved in platelet–leukocyte interactions.

In this study, we tested the relationships between platelet size indexes and RP percentage (RP %) and platelet function (exposure of activated GP IIb-IIIa and P-selectin) in cardiovascular patients with coronary heart disease (CHD) or ACS, receiving dual antiplatelet therapy, acetylsalicylic acid (ASA, an inhibitor of cyclooxygenase and thromboxane A2 synthesis) in combination with clopidogrel or ticagrelor (antagonists of P2Y12 ADP receptors), and in the control group consisted of patients with risk factors for CHD, but no CHD/ACS, and free of antiplatelet drugs.

## 2. Materials and Methods

### 2.1. Blood Collection and Patients

Blood was collected from CHD patients receiving ASA (75–100 mg/daily) + clopidogrel (75 mg/daily) (n = 55), ACS patients receiving ASA (75–100 mg/daily) + ticagrelor (90 mg × 2/daily) (n = 95), and from patients of the control group (n = 66) with no diagnosed CHD/ACS and free of antiplatelet drugs, but with the risk factors for CHD (hypertension, diabetes mellitus, hypercholesterolemia). In ACS patients, blood was collected on days 3–5 after the onset of the disease. Main characteristics of the control, CHD, and ACS groups are given in Appendix A, Table A1. All groups were comparable in age, while the number of males was greater in CHD and ACS groups. Control group included slightly fewer patients with hypertension and about the same number of patients with diabetes mellitus and hypercholesterolemia in comparison with CHD and ACS groups. There were slightly fewer males and slightly more patients with hypercholesterolemia in CHD in comparison with ACS. All participants were treated or monitored at the Chazov National Medical Research Center of Cardiology and signed informed consent for participation in the study. The study was approved by the Ethics Committee of Chazov National Medical Research Center of Cardiology (protocol # 279, 25 April 2022).

Blood was taken from the antecubital vein using 18 g needles into 5% EDTA and 3.8% sodium citrate at a blood/anticoagulant ratio 9/1.

### 2.2. Platelet Count, Mean Platelet Volume (MPV), and Platelet Large Cell Ratio (PLC-R)

Platelet count, MPV, and PLC-R were measured in EDTA-anticoagulated blood in an Abacus Junior B hematological analyzer (Diatron, Budapest, Hungary). Patients with thrombocytopenia (platelet count < 100 × 109/L) were excluded from the study.

### 2.3. Reticulated Platelets (RP)

RP were identified in EDTA-anticoagulated blood using thiazole orange (TO) as described earlier [19]. A total of 5 µL of whole blood was added to 1 mL of BD FACS Flow reagent (BD Bioscience, San Jose, CA) supplemented with 5 µL of CD42b-APC (BD Biosciences, San Jose, CA, USA) and 50 µL of 10 µg/mL TO solution. Control samples contained no TO. Samples were incubated (30 min, room temperature), cells were spinned down (2500 g, 3 min), and the pellet was resuspended in BD FACS Flow reagent. Platelets were analyzed in a BD FACSCantoTM II flow cytometer and BD FACS DivaTM Software v8.0.1. (BD Biosciences, San Jose, CA, USA) was used for data evaluation. Platelets were identified (gated) by size and CD42b-APC staining (see Supplement Figure S1); 10,000 platelets were analyzed in each sample. Fluorescence of TO positive platelets (RP) was higher than fluorescence of >99% platelets in the control sample (no TO). RP percentage (RP %) in the whole platelet population was estimated.

### 2.4. Platelet Forward Scattering (FSC)

Platelet FSC mean values were determined by flow cytometry in the same sample as RP (see above) and expressed in arbitrary units (a.u.).

### 2.5. Platelet Function

Platelet function was evaluated in citrated blood using flow cytometry. Exposure of platelet activation markers, and activated GP IIb-IIIa and P-selectin (binding of PAC-1 and CD62P antibodies respectively), was measured as described earlier [19]. Blood was diluted 6 times with Tyrode/HEPES solution with no CaCl_2_ (137 mM NaCl, 2.7 mM KCl, 0.36 mM NaH_2_PO_4_, 0.1% dextrose, 5 mM HEPES, pH 7.35, 1 mM MgCl_2_), containing 0.35% BSA. A total of 60 µL of diluted blood was supplemented with 3 µL of CD42b-APC and either with 10 µL of PAC-1-FITC (BD Biosciences, San Jose, CA, USA), 5 µL of CD62P-FITC (IMTEK, Moscow, Russia), or 5 µL of mouse IgG-FITC (IMTEK, Moscow, Russia). In the control group, platelets were not activated or activated with thrombin receptor activating peptide (TRAP, sequence SFLLRN, kindly provided by Mikhail V. Ovchinnikov, the Chazov National Medical Research Center of Cardiology) 10 µM or ADP (AppliChem GmbH, Darmstadt, Germany) 20, 5, or 2.5 µM; in CHD/ACS groups, platelets were not activated or activated with TRAP 10 µM and ADP 20 and 5 µM in the absence or presence epinephrine (AppliChem GmbH, Darmstadt, Germany) 20 µM. After an incubation of 15 min, 37 °C in the dark, samples with antibodies were fixed with 1% paraformaldehyde (final concentration) and diluted with BD FACS Flow reagent. Platelets were analyzed in a BD FACSCantoTM II flow cytometer and BD FACS DivaTM Software was used for data evaluation. Platelets were identified (gated) by size and CD42b-APC staining (see Supplement Figure S1); 10,000 platelets were analyzed in each sample. Mean fluorescence intensity (MFI, a.u.) for PAC-1-FITC and CD62P-FITC binding and the percentages of PAC-1 and CD62P positive platelets (PAC-1+ and CD62P+) were evaluated. Fluorescence of the PAC-1+ and CD62P+ platelets was higher than the fluorescence of 95% platelets in control samples (non-activated platelets for PAC-1 and non-activated platelets with mouse IgG-FITC for CD62P).

### 2.6. Interleukin 6 (IL6)

IL6 was measured in plasma prepared from EDTA-anticoagulated blood (1500 g, 15 min × 2) using DuoSet Human IL6 kit (R&D Systems, Minneapolis, MN, USA) according to the manufacturer’s instruction.

### 2.7. Statistics

Statistical analysis was performed using Statistica 12 software (Stat. Soft., Tulsa, OK, USA). Most variables fitted normal distribution (Shapiro–Wilk test). Data were presented as means ± standard deviations (SDs). Intergroup differences were estimated using Student’s *t*-test for means for comparison of quantitative variables and the Chi-square test for comparison of qualitative variables. Correlations were assessed using Pearson test.

## 3. Results

Platelet size indexes (MPV, PLC-R, and FSC), RP %, and the exposure of activated GP IIb-IIIa and P-selectin after platelet activation with different agonists were measured in the control (without CHD/ACS, receiving no antiplatelet drugs), CHD (ASA + clopidogrel), and ASC (ASA + ticagrelor) groups of patients (for patient characteristics, see Appendix A, Table A1). Intergroup differences for platelet size indexes and RP % were insignificant, reaching statistical significance only for MPV between the CHD and ACS groups (Table 1). In all groups, the correlations between different platelet size indexes were strong and significant (r > 0.5, *p* < 0.001), although MPV and P-LCR were measured in a hematological analyzer and FSC was measured in a flow cytometer (Table 1). Mostly strong and highly significant correlations were also detected between the RP % and size indexes (r > 0.5, *p* < 0.001). Weaker, but still significant, correlations were found only between RP % and MPV in the CHD and ACS groups (r = 0.348 and r = 0.278, respectively, *p* < 0.01) (Table 1).

**Figure 1:**
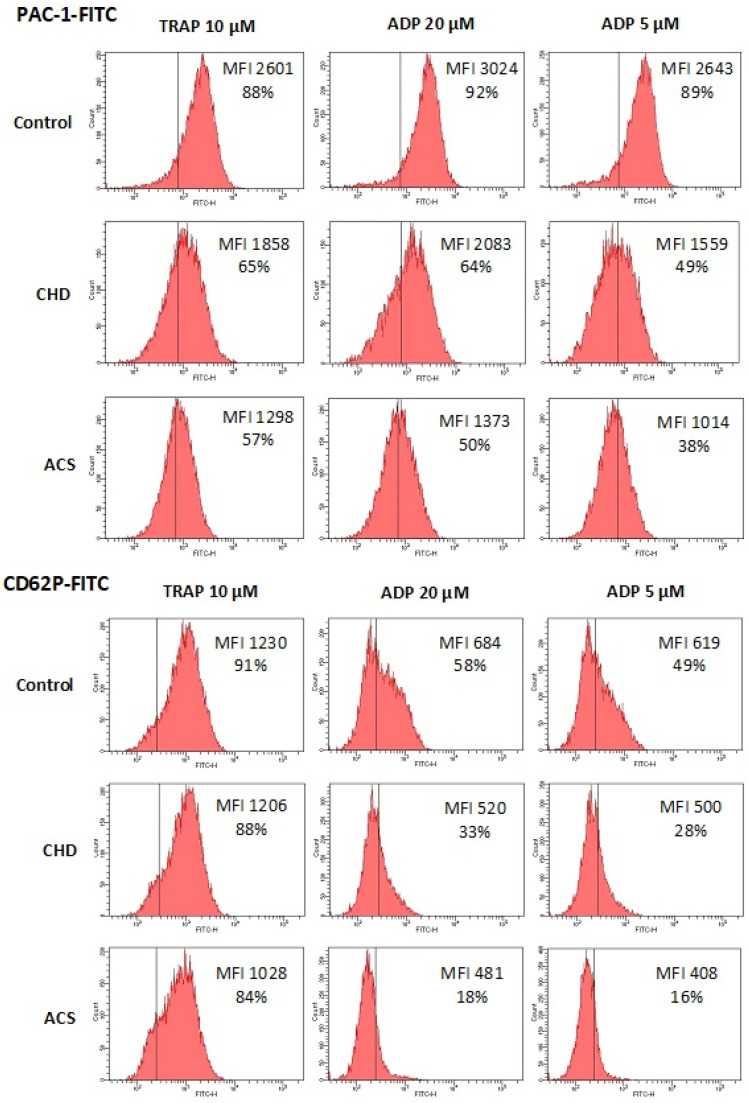
Platelet activity in the control (no antiplatelet drugs), CHD (ASA + clopidogrel), and ACS (ASA + ticagrelor) groups. Flow cytometry. X-axis—“Count”, Y-axis—“FITC” fluorescence. Exposure of activated GP IIb-IIIa determined by PAC-1-FITC binding (upper panel) and exposure of P-selectin determined by CD62P-FITC binding (lower panel). Platelets were activated by TRAP 10 µM, and ADP 20 and 5 µM. Vertical line: negative control level (95% threshold in samples with no agonists). MFI and % of PAC-1+ and CD62P+ platelets are shown. X-axis—“Count”, Y-axis—“FITC-H” fluorescence. Representative examples.

Platelet function was assessed by the exposure of two activation markers: activated GP IIb-IIIa (PAC-1 antibody binding) and P-selectin (CD62P antibody binding). In the control group (no antiplatelet drugs), platelets were activated by TRAP 10 µM, and ADP 20, 5, and 2.5 µM. In the CHD and ACS groups receiving strong dual antiplatelet therapy, we omitted the lowest ADP dose (2.5 µM) and all other agonists were added not only alone, but also in combination with 20 µM epinephrine, which potentiates their effects on platelet activation (see Table 2). The exposure of activated GP IIb-IIIa and P selectin in response to the same agonists (TRAP 10 µM, and ADP 20, 5 µM) was expectedly lower in the CHD and ACS groups in comparison with the control group due to the applied antiplatelet therapy (all differences were significant except for TRAP 10 µM, CD62P+, % in the CHD group) (Figure 1 and Table 2). A decrease in platelet activity was greater in the ACS group in comparison to the CHD group since ticagrelor is a more potent P2Y12 antagonist than clopidogrel, although differences were not significant for some activation indexes (predominantly for the most powerful agonist—TRAP 10 µM + Epinephrine, 20 µM) (Figure 1 and Table 2). No signs of platelet preactivation and no differences between groups were detected in samples without agonists (Table 2).

In the control group, (no antiplatelet drugs) increased platelet size (assessed by all indexes) and increased RP % were associated with the increased exposure of both markers, and activated GP IIb-IIIa and P-selectin (PAC-1-FITC and CD62-FITC MFI levels) after platelet activation with all applied agonists (TRAP 10 µM, ADP 20, 5 and 2.5 µM). Moderate to high correlations were detected (r from 0.300 to 0.551) with high significance (*p* mainly < 0.001 or <0.01) (Table 3; for examples of positive correlation plots, see Supplement Figure S2).

In the CHD (ASA + clopidogrel) and ACS (ASA + ticagrelor) groups, platelets were activated by TRAP 10 µM, and ADP 20 and 5 µM ± epinephrine 20 µM. In many cases, significant correlations between platelet size indexes, RP %, and activated GP IIb-IIIa and P-selectin (PAC-1-FITC and CD62-FITC MFI levels) were detected regardless of the effect of strong antiplatelet drugs (some r values > 0.5) (Table 4, for examples of positive correlation plots, —see Supplement Figure S2). More frequently, significant correlations were established for activated GP IIb-III than for P-selectin, particularly after platelet activation with ADP or ADP + epinephrine. Most rarely, significant correlations were detected between MPV and both activation markers (Table 4; for examples of correlation plots, see Supplement Figure S2).

Plasma IL6 content was measured in the majority of patients in the control (n = 45), CHD (n = 44), and ACS groups (n = 82). All mean values varied in the range of 40–50 pg/mL and did not differ significantly between groups. In the control group, platelet size (all indexes) and RP % correlated with IL6 content: r—0.641 (*p* < 0.001), 0.398 (*p* < 0.01), 0.520 (*p* < 0.001), and 0.430 (*p* < 0.01) for MPV, PLC-R, FSC, and RP %, respectively. These correlations were determined by the presence of several patients with extremely high IL6 levels (>100 pg/mL) (for an example of correlation plot (MPV versus IL6), see Supplement Figure S3), and their exclusion made correlations weak and not significant. Patients with high IL6 content were identified in the CHD and ACS groups as well, but no correlations were detected between platelet size indexes, RP %, and IL6 content in those groups (r < 0.1 everywhere).

## 4. Discussion

We examined the effects of two individual phenotypic factors, namely platelet size, assessed by different indexes (MPV, PLC-R, and FSC) and RP content (% in the entire platelet population) on platelet functional activity in cardiovascular patients. Previously, we revealed a direct association between platelet size indexes and RP %, and the exposure of activated GP IIb-IIIa and P-selectin in healthy volunteers free of drugs affecting platelet function [19]. In the present study, we confirmed these results in a slightly different group, also free of antiplatelet drugs. This group (the control), consisted of patients with risk factors but without diagnosed CHD. Two other groups of patients with CHD and ACS received strong dual antiplatelet therapy, with ASA + clopidogrel and ASA + ticagrelor, respectively. Antiplatelet drugs inhibited platelet function, with the inhibitory effect being potentially influenced by the individual variations of pharmacodynamics and/or pharmacokinetics (particularly for clopidogrel when platelets are affected by active clopidogrel metabolite produced in the liver). However, even under these conditions, we detected the influence of platelet size and RP % on platelet functional activity. Significant correlations were detected less frequently than in the control group, but some correlations were still strong (r > 0.5) and highly significant. Guthikonda et al. [14] analyzed GP IIb-IIIa and P-selectin exposure induced by 10 µM ADP in CHD patients receiving ASA + clopidogrel who were stratified into tertiles according to RP %. They reported differences for activated GP IIb-IIIa but not P-selectin exposure between tertiles with high and low RP content. Here, we failed to obtain correlations of the ADP-induced exposure of both markers with RP % in the comparable CHD group (ASA + clopidogrel), and the cause of the discrepancy in results on activated GP IIb-IIIa is unclear. However, we revealed significant correlations of activated GP IIb-IIIa and RP % in the ACS group (ASA + ticagrelor), confirming the influence of RP content on this reaction in patients receiving strong antiplatelet therapy. Most authors studying the effects of platelet size and RP content on routine platelet aggregation response reported positive correlations in patients receiving ASA + clopidogrel but not ASA + ticagrelor [8,10,11]. Very low ADP-induced aggregation rates in patients treated with ticagrelor (in some cases, close to zero level) which is more potent blocker of P2Y12 ADP receptor than clopidogrel, may account for the lack of the observed effects. We have demonstrated correlations of platelet size indexes and RP % with ADP-(as well as TRAP-) induced activated GP IIb-IIIa exposure (a reaction preceding fibrinogen binding and aggregation) in ACS patients treated with ASA + ticagrelor. Presumably, this is due to the relatively high levels of PAC-1 antibody binding to activated GP IIb-IIIa, even under the action of ASA + ticagrelor (at least far from zero level), particularly in the presence of epinephrine. For CD62P exposure, significant interactions of size indexes and RP % in both groups were obtained mainly in the TRAP-activated platelets. Thus, the data obtained in our study indicated that increased platelet size and RP content can diminish the effects on platelet function, even in strong dual therapy such as ASA + ticagrelor.

High platelet activity in patients with increased platelet size and RP content could explain why the corresponding laboratory indexes (MPV and RP %) serve as moderate risk factors of thrombotic events in cardiovascular patients, including those receiving dual antiplatelet therapy. The validity of these factors was estimated in separate clinical studies and meta-analysis as well [9,10,11,25,26].

Both platelet size and RP content reflects the intensity of platelet production by megakaryocytes in the bone marrow. Presumably, in some patients with accelerated thrombocytosis, the release of “young” (reticulated) and large platelets is enhanced. Thrombopoietin and IL6 are the two major regulators of megakaryocyte maturation and functioning [27,28]. In two studies, including our own, modest, although significant associations were reported between increased platelet size (assessed by the MPV index) and the amount of TPO in the blood plasma of cardiovascular patients [16,29]. In the present investigation, we attempted to access interactions of size indexes and RP % with IL6 levels. We established significant correlations only in the control but not in the CHD and ACS groups. Thus, our data do not allow to suggest a significant role of increased IL6 in the regulation of platelet production, platelet size, and RP content, at least in CHD and ACS patients.

## 5. Conclusions

Our data have shown that increased platelet size and increased RP content are associated with increased platelet activity in cardiovascular patients free of antiplatelet drugs and receiving dual antiplatelet therapy.

## Figures and Tables

**Table 1:** Platelet size indexes and RP % in the control (no antiplatelet drugs) (n = 66), CHD (ASA + clopidogrel) (n = 55), and ACS (ASA + ticagrelor) (n = 95) groups.

	MPV, fl	PLC-R %	FSC, a.u.	RP %
Control	8.7 ± 1.1	27.2 ± 7.6	20,320 ± 4556	12.8 ± 5.5
r (MPV)	-	0.904	0.779	0.506
r (PLC-R)			0.841	0.537
r (FSC)				0.694
CHD	8.7 ± 0.8	27.8 ± 6.1	20,144 ± 4997	11.6 ± 4.1
r (MPV)	-	0.891	0.538	0.348
r (PLC-R)			0.642	0.480
r (FSC)				0.467
ACS	8.4 ± 0.8 *	25.9 ± 6.6	19,753 ± 4060	12.2 ± 5.2
r (MPV)	-	0.922	0.628	0.278
r (PLC-R)			0.779	0.486
r (FSC)				0.650

Means ± SD and r (coefficients of correlations) with indicated indexes (MPV, PLC-R, and FSC) are presented. * *p* < 0.05—significance of differences from “CHD” group. Significance of correlations at r > 0.4—*p* < 0.001; at r < 0.4—*p* < 0.01.

**Table 2:** Platelet activity in the control (no antiplatelet drugs) (n = 66), CHD (ASA + clopidogrel) (n = 55), and ACS (ASA + ticagrelol) (n = 95) groups. Exposure of activated GP IIb-IIIa (PAC-1 binding) and P-selectin (CD62P binding).

Agonist	Index	Control	CHD *p* (Control) < 0.001 ^a^	ACS *p* (Control) < 0.001 ^b^
**Activated GP IIb-IIIa (PAC-1 binding)**				
No agonists	MFI, a.u	365 ± 84	373 ± 90	346 ± 84
TRAP 10 µM	MFI, a.u.	2830 ± 1124	1802 ± 718	1405 ± 462 ***
	PAC-1+, %	88 ± 7	68 ± 15	59 ± 12 ***
TRAP 10 µM Epi 20 µM	MFI, a.u	n.d.	2784 ± 971	2531 ± 704
	PAC-1+, %	n.d.	87 ± 8	85 ± 7
ADP 20 µM	MFI, a.u	3186 ± 1184	1934 ± 780	1425 ± 507 ***
	PAC-1+, %	90 ± 5	61 ± 19	48 ± 12 ***
ADP 20 µM Epi 20 µM	MFI, a.u	n.d.	2883 ± 1008	2572 ± 805 *
	PAC-1+, %	n.d.	86 ± 9	83 ± 8
ADP, 5 µM	MFI, a.u	2940 ± 1103	1705 ± 694	1237 ± 445 ***
	PAC-1+, %	87 ± 6	55 ± 20	37 ± 11 ***
ADP 5 µM Epi 20 µM	MFI, a.u	n.d.	2686 ± 989	2280 ± 725 **
	PAC-1+, %	n.d.	84 ± 10	78 ± 10 ***
ADP 2.5 µM	MFI, a.u	2690 ± 1044	n.d.	n.d.
	PAC-1+, %	83 ± 8	n.d.	n.d.
**P-selectin (CD62P binding)**				
No agonists	MFI, a.u.	338 ± 143	320 ± 128	348 ± 138
	CD62P+, %	8 ± 4	10 ± 6	9 ± 5
TRAP 10 µM	MFI, a.u.	1443 ± 455	1277 ± 334	1166 ± 317 *
	CD62P+, %	90 ± 8	89 ± 6	87 ± 8
TRAP 10 µM Epi 20 µM	MFI, a.u	n.d.	1484 ± 373	1491 ± 381
	CD62P+, %	n.d	93 ± 4	92 ± 5
ADP 20 µM	MFI, a.u	723 ± 202	537 ± 177	427 ± 97 ***
	CD62P+, %	57 ± 15	35 ± 17	21 ± 10 ***
ADP 20 µM Epi, 20 µM	MFI, a.u	n.d.	784 ± 265	648 ± 171 ***
	CD62P, %	n.d.	61 ± 17	52 ± 16 ***
ADP 5 µM	MFI, a.u	643 ± 179	496 ± 153	393 ± 84 ***
	CD62P+, %	50 ± 16	31 ± 16	18 ± 10 ***
ADP 5 µM Epi 20 µM	MFI, a.u	n.d.	720 ± 238	594 ± 149 ***
	CD62P+, %	n.d.	57 ± 18	47 ± 16 ***
ADP 2.5 µM	MFI, a.u	594 ± 156	n.d.	n.d.
	CD62P+, %	46 ± 15	n.d.	n.d.

Epi—epinephrine. ^a^ All differences between the CHD and control groups were highly significant (*p* < 0.001) except “No agonists” (not significant), TRAP 10 µM, CD62P MFI, a.u. (*p* < 0.05), and TRAP 10 µM, CD62P+, % (not significant). ^b^ All differences between the ACS and control groups were highly significant (*p* < 0.001) except “No agonists” (not significant) and TRAP 10 µM, CD62P+, % (*p* < 0.05). * *p* < 0.05, ** *p* < 0.01, *** *p* < 0.001—significance of differences between the CHD and ACS groups. n.d.—not determined.

**Table 3:** Interactions of activated GP IIb-IIIa (PAC-1 binding) and P-selectin (CD62P binding) exposure with platelet size indexes (MPV, P-LCR, FSC) and RP %. Control group (no antiplatelet drugs) (n = 66).

Agonist	MPV	P-LCR	FSC	RP %
**PAC-1, MFI**				
TRAP 10 µM	*0.356* **	*0.385* **	*0.412* ***	*0.450* ***
ADP 20 µM	*0.460* ***	*0.473* ***	*0.528* ***	*0.536* ***
ADP 5 µM	*0.450* ***	*0.471* ***	*0.492* ***	*0.520* ***
ADP 2.5 µM	*0.471* ***	*0.473* ***	*0.485* ***	*0.519* ***
**CD62P, MFI**				
TRAP, 10 µM	*0.320* **	*0.458* ***	*0.546* ***	*0.557* ***
ADP 20 µM	*0.300* *	*0.321* **	*0.448* ***	*0.546* ***
ADP 5 µM	*0.330* **	*0.330* **	*0.431* ***	*0.551* ***
ADP 2.5 µM	*0.321* **	*0.385* **	*0.413* ***	*0.547* ***

Coefficients of correlations (r) are presented. * *p* < 0.05, ** *p* < 0.01, *** *p* < 0.001—significance of correlations. Significant correlations are shown in italics (all correlations in this table).

**Table 4:** Interactions of activated GP IIb-IIIa (PAC-1 binding) and P-selectin (CD62P binding) exposure with platelet size indexes (MPV, P-LCR, FSC) and RP %. CHD (ASA + clopidogrel) (n = 55) and ACS (ASA + ticagrelor) (n = 95) groups.

**CHD (ASA + Clopidogrel)**				
**Agonist**	**MPV**	**P-LCR**	**FSC**	**RP %**
**PAC-1, MFI**				
TRAP 10 µM	*0.290* *	*0.484* ***	*0.410* **	0.141
TRAP 10 µM + Epi 20 µM	0.233	*0.475* ***	*0.370* **	0.235
ADP 20 µM	0.217	*0.447* ***	*0.360* **	0.163
ADP 20 µM + Epi 20 µM	0.190	*0.445* ***	*0.319* *	0.186
ADP 5 µM	0.208	*0.445* ***	*0.376* **	0.176
ADP 5 µM + Epi 20 µM	0.184	*0.437* ***	*0.316* *	0.151
**CD62P, MFI**				
TRAP 10 µM	*0.264* *	*0.489* ***	*0.477* ***	*0.408* **
TRAP 10 µM + Epi 20 µM	0.235	*0.495* ***	*0.474* ***	*0.457* ***
ADP 20 µM	0.209	0.201	0.221	0.097
ADP 20 µM + Epi 20 µM	0.196	0.233	0.218	0.151
ADP 5 µM	0.216	0.198	0.248	0.077
ADP 5 µM + Epi 20 µM	0.203	0.224	0.255	0.144
**ACS (ASA + Ticagrelor)**				
**Agonist**	**MPV**	**P-LCR**	**FSC**	**RP %**
**PAC-1, MFI**				
TRAP 10 µM	0.073	*0.261* *	*0.507* ***	*0.459* ***
TRAP 10 µM + Epi 20 µM	0.004	0.172	*0.376* ***	*0.319* **
ADP 20 µM	0.119	*0.283* **	*0.504* ***	*0.451* ***
ADP 20 µM + Epi 20 µM	0.052	*0.235* *	*0.440* ***	*0.411* ***
ADP 5 µM	0.094	*0.282* **	*0.527* ***	*0.477* ***
ADP 5 µM + Epi 20 µM	0.052	*0.234* *	*0.447* ***	*0.388* ***
**CD62P, MFI**				
TRAP 10 µM	0.156	*0.369* ***	*0.487* ***	*0.600* ***
TRAP 10 µM + Epi 20 µM	0.161	*0.418* ***	*0.541* ***	*0.605* ***
ADP 20 µM	*0.244* *	0.116	0.052	0.061
ADP 20 µM + Epi 20 µM	0.175	0.171	0.190	0.157
ADP 5 µM	*0.270* *	0.142	0.074	0.058
ADP 5 µM + Epi 20 µM	*0.209* *	0.183	0.146	0.124.

Epi—epinephrine. Coefficients of correlations (r) are presented. * *p* < 0.05, ** *p* < 0.01, *** *p* < 0.001—significance of correlations. Significant correlations are shown in italics.

## Data Availability

Data are contained within the article and supplementary materials.

## Supplementary Materials

The following supporting information can be downloaded at: https://www.mdpi.com/article/10.3390/cells13201712/s1, Figure S1: Platelet gating in the whole blood. Flow cytometry; Figure S2: Examples of correlation plots for interactions of platelet function (exposure of activated GP IIb-IIIa, PAC-1, MFI and P-selectin, CD62, MFI) with platelet size indexes (MPV, PLC-R and FSC) and RP % in the control (no antiplatelet drugs), CHD (ASA + clopidogrel) and ACS (ASA + ticagrelor) groups; Figure S3: Correlation of MPV and plasma IL-6 in the control group.

## Appendix A

**Table A1:** Main characteristics of the control (no antiplatelet drugs), CHD (ASA + clopidogrel), and ACS (ASA + ticagrelor) groups of patients.

	Control	CHD	ACS
n	66	55	95 ^a^
Age (mean ± SD), years	62 ± 13	65 ± 11	62 ± 10 p(CHD) = 0.052
Male/female, n/n	30/36	37/18 *	78/17 *** p(CHD) = 0.038
Hypertension, n (%)	47 (71%)	51 (93%) **	84 (88%) ** p(CHD) = 0.398
Diabetes mellitus, n (%)	10 (15%)	15 (27%)	22 (23%) p(CHD) = 0.573
Hypercholesterolemia, n (%)	22 (33%)	27 (49%)	30 (32%) p(CHD) = 0.033

^a^ Myocardial infarction, n = 89; unstable angina, n = 6. * *p* < 0.05; ** *p* < 0.01, *** *p* < 0.001—significance of differences from “Control” group; p(CHD)—significance of differences from “CHD” group.
