# Supplementary material for: Platelet Function, Platelet Size and Content of Reticulated Platelets: Interactions in Patients Receiving Dual Antiplatelet Therapy
Source: Cells. 2024 Oct 16;13(20):1712. doi: 10.3390/cells13201712 (PMC11506637; doi:10.3390/cells13201712)
Supplement: Supplementary file 1 [file cells-13-01712-s001.zip › cells-3208861-supplementary.pdf]

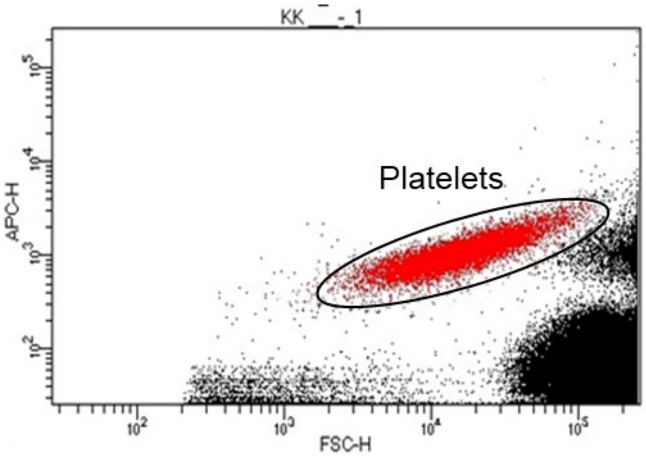

Figure S1. Platelet gating in the whole blood. Flow cytometry. Platelets (red color) were gated according to their size and staining with CD42b-APC.

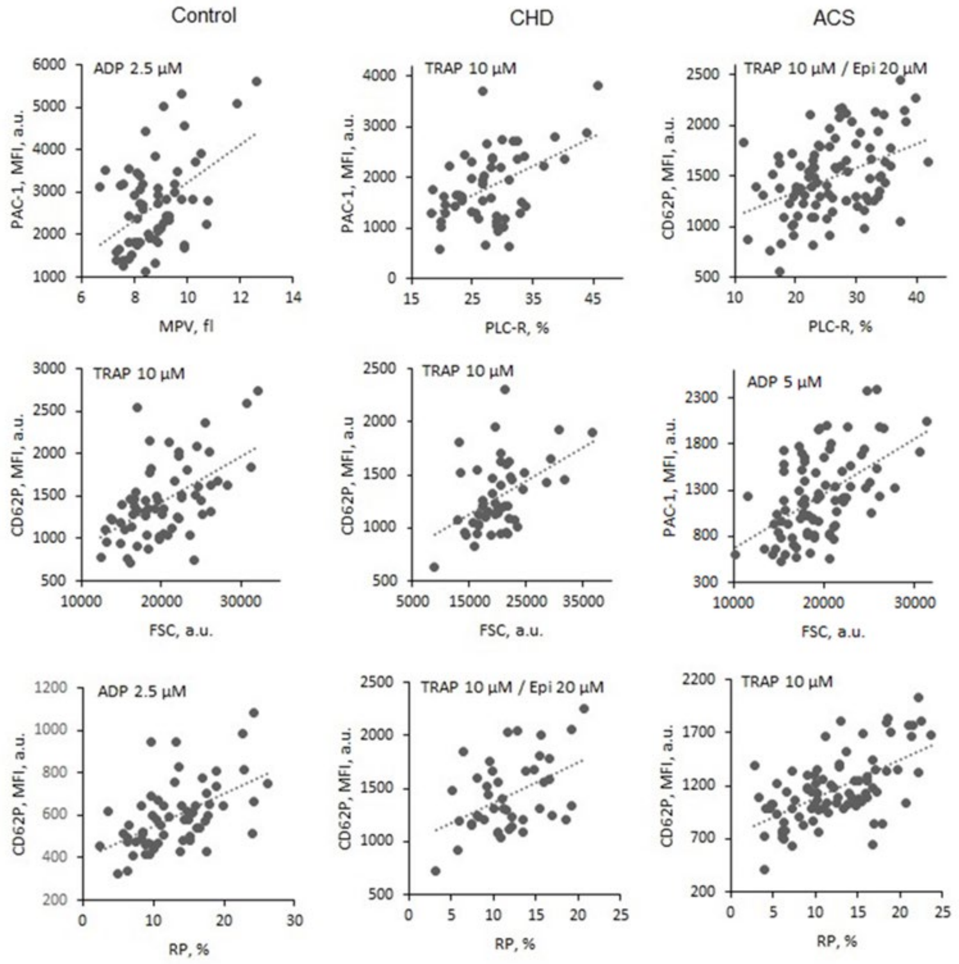

Figure S2. Examples of correlation plots for interactions of platelet function (exposure of activated GP IIb-IIIa, PAC-1, MFI and P-selectin, CD62, MFI) with platelet size indexes (MPV, PLC-R and FSC) and RP, % in the control (no antiplatelet drugs), CHD (ASA + clopidogrel) and ACS (ASA + ticagrelor) groups. Agonists used for platelet activation are indicated in each panel (Epi – epinephrine). Compared indexes are indicated at the axes legends. Statistical data – see Tables 3 and 4.

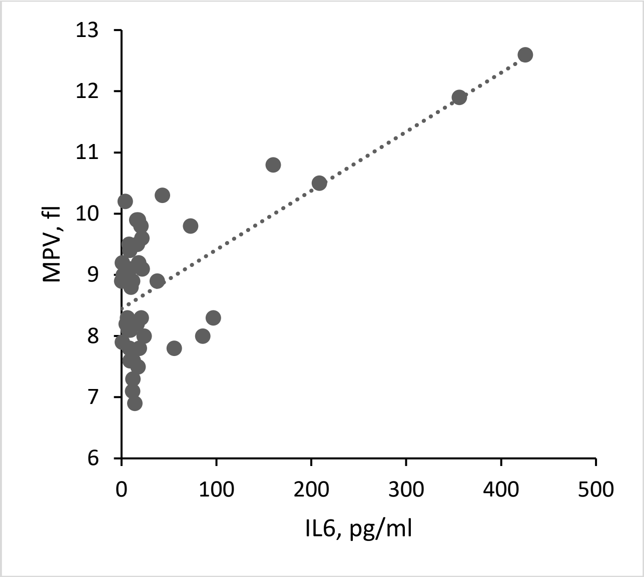

Figure S3. Correlation of MPV and plasma IL-6 in the control group; r = 0,641, p < 0.001 (n = 45).
